# Development, validation and application of a device to measure e-cigarette users’ puffing topography

**DOI:** 10.1038/srep35071

**Published:** 2016-10-10

**Authors:** Anthony Cunningham, Sandra Slayford, Carl Vas, Jodie Gee, Sandra Costigan, Krishna Prasad

**Affiliations:** 1 Research and Development, British American Tobacco (Investments) Ltd., Regents Park Road, Southampton SO15 8TL, United Kingdom; 2 Nicoventures Holdings Ltd., Globe House, 1 Water Street, London WC2R 3LA, United Kingdom

## Abstract

With the rapidly rising popularity and substantial evolution of electronic cigarettes (e-cigarettes) in the past 5–6 years, how these devices are used by vapers and consumers’ exposure to aerosol emissions need to be understood. We used puffing topography to measure directly product use. We adapted a cigarette puffing topography device for use with e-cigarettes. We performed validation using air and e-cigarette aerosol under multiple regimes. Consumer puffing topography was measured for 60 vapers provided with rechargeable “cig-a-like” or larger button-activated e-cigarettes, to use ad-libitum in two sessions. Under all regimes, air puff volumes were within 1 mL of the target and aerosol volumes within 5 mL for all device types, serving to validate the device. Vapers’ mean puff durations (2.0 s and 2.2 s) were similar with both types of e-cigarette, but mean puff volumes (52.2 mL and 83.0 mL) and mean inter-puff intervals (23.2 s and 29.3 s) differed significantly. The differing data show that product characteristics influence puffing topography and, therefore, the results obtained from a given e-cigarette might not read across to other products. Understanding the factors that affect puffing topography will be important for standardising testing protocols for e-cigarette emissions.

The use and awareness of electronic cigarettes (e-cigarettes, also known as electronic nicotine delivery systems or ENDS) has increased rapidly in the past 5–6 years^1^,^2^,^3^. E-cigarettes represent an alternative to conventional combustible cigarettes, and generally work by vaporising a solution containing nicotine to produce aerosol^4^,^5^. E-cigarettes have seen rapid development from first generation “cig-a-like” disposable devices through to larger, more powerful, customisable devices, offering users a range of devices with varying nicotine delivery^6^. There is much scientific debate on the role of e-cigarettes in tobacco harm reduction^7^,^8^, and the exposure of users to emissions from e-cigarettes is an important factor in this debate. Assessment of user puffing topography is one route to measuring this information.

Puffing topography involves the measurement of puff volumes, durations, numbers, flow rates and intervals. Topography data can be obtained in part by observational methods, such as video recording^9^, or more thoroughly through the use of puffing flow measurement devices^10^,^11^. Puff duration has also been measured in button-activated e-cigarettes by measuring the length of time the heating element is activated by the user^12^,^13^. Puffing topography data have been extensively studied for users of combustible cigarettes, and the impact on smoking experience has been widely reported^9^,^11^,^14^. These studies generally show that puffing topography devices do not significantly alter smokers’ exposure, although some reductions in the intensity of smoking attributes have been reported. Equivalent puffing topography data for users of e-cigarettes are limited, but initial studies with video recordings suggest increased puff durations compared with cigarette smoking^15^,^16^. Studies have also measured e-cigarette users’ puffing topography with a number of commercial and non-commercial puffing topography devices^17^,^18^,^19^,^20^, although reliability issues and limitations in recording more than 43 puffs have been reported for some commercial devices.

We report the validation of a puffing topography device previously used with tobacco cigarettes and modified for use with e-cigarettes to address previously reported challenges in measurement^19^. The first feature to address was avoiding or limiting condensation and deposition of the e-cigarette aerosol within the topography device, which can affect the accuracy of measurements. Second, was to ensure that the device could accurately record a sufficiently wide range of puff durations and volumes to accurately reflect actual use, as substantial variability in e-cigarette puffing behaviour is reported in the literature. The modified topography device has been used to increase understanding of how e-cigarette use varies between individuals and devices, which is an important step on the road to relevant standards for laboratory aerosol emission tests and assessing user exposure to the constituents of e-cigarette emissions either on the market or pre-launch.

## Methods

### Topography Device Development

The e-cigarette topography device (Patent pending; UK application number 1420649.4 [unpublished]) was a modified version of the non-commercial SA7 topography device, which senses flow-induced pressure differentials across an orifice plate every 40 ms^21^. Full details of the device design, operation and data generation for the SA7 device have been reported elsewhere^21^. The modifications involved moving two pressure tubes connected to the pressure transducer towards the top of the topography head to reduce the condensation and build-up of e-cigarette aerosol within the tubing, which results from increased aerosol concentrations and viscosity compared with that from combustible cigarettes. Additionally, a removable cap was incorporated into the topography head to allow easy cleaning of the orifice plates (between users), and the addition of a spigot (to act as a spacer) to reduce the “jetting” effect that occurs when the e-cigarette aerosol passes through the pressure orifice, as preliminary experiments indicated that this led to inaccurate puff volume determinations. The introduction of the spigot allows the fine jet of aerosol to disperse prior to reaching the pressure orifice, maintaining the relationship between differential pressure and flow through the device. The spigot is connected to a short length of flexible tubing which allows the topography device to be used with the various mouthpiece designs used across e-cigarettes. Finally, a supporting bracket was added to the topography head to aid use of the device with larger e-cigarette models, which often require the user to activate a button before puffing. The modified SA7 head is used in conjunction with a data acquisition and transfer unit and a laptop computer (see Supplementary Fig. S1)^21^.

### Topography Device Validation

Pressure calibration of the topography device was achieved using a calibrated pressure meter (C9551, Comark, Norwich, UK) at flow rates 17.5 mL/s and 120 mL/s to generate pressures in the region of 100 mmWg and 660 mmWg, respectively. Flow rate calibration was completed at 33 flow rates from 2 mL/s to 120 mL/s, using a modified A14 syringe driver (Borgwaldt KC, Hamburg, Germany). A Tri-City smoke machine (MBC2000, Borgwaldt) was used to draw known volumes of air under four puff regimes (see Supplementary Table S1), with a pressure drop (15 mmWG) placed in-line between the topography head and the smoking machine to generate the draw resistance required to trigger data recording by the data acquisition unit. Puff volumes recorded by the modified topography head were compared with those obtained via a soap bubble burette. Puff durations were compared with values obtained with an unmodified SA7 topography head, as those measured by topography devices are typically shorter than the pre-set smoke machine values^21^. Each of the four puffing regimes was tested in triplicate, with use of a variety of puff profiles - sine, rectangle, rectangle with an incline and decline, triangle and early triangle profiles.

Additional validation of the modified topography device to accurately measure flow rates of the aerosol produced by e-cigarettes was completed. Three puffing regimes with rectangle puff profiles (see Supplementary Table S2) were used to puff a disposable and a rechargeable “cig-a-like” e-cigarette until battery exhaustion and a refillable modular e-cigarette to a maximum of 150 puffs (see product section for details). Regimes 1 and 2 were generated with the Tri-City smoke machine, whereas the Borgwaldt A14 syringe driver was used to generate the more intense regime 3, due to the maximum puff volume of 80 mL possible with the Tri-City smoke machine. Each regime was tested in triplicate for each e-cigarette device type. Puff volumes were compared with known volumes of air drawn by the smoke machine.

Prior experiments conducted to test the suitability of the unmodified device to accurately measure flow rates of the aerosol from a disposable e-cigarette involved pressure and flow rate calibration using the procedure described above followed by puff volume measurements using a disposable e-cigarette at four volumes from 25 mL to 100 mL (to represent a clean system). Four measurements at each volume were recorded. To simulate repeated use of the topography device, the puff volume measurements were repeated following 100 puffs on the disposable e-cigarette using of puffing regime of 80 mL volume, 3 s duration and 30 s interval.

### Study Participants and Ethics Statement

Participants were recruited by a third party agency. Eligible participants were aged between 21 and 64 years and had used either a rechargeable cartridge-based or refillable tank-based (modular) e-cigarette on two or more days per week for at least 1 month, including dual users of e-cigarettes and tobacco products. Women were excluded if they reported being pregnant or breastfeeding. Participants who met the screening criteria were briefed on the study protocol before giving their written informed consent to participate in the study. The protocol and Informed Consent Form were approved by the Human Research Committee (HRC), the internal ethics committee of British American Tobacco. BAT’s HRC reviews all studies involving human subjects or human tissue samples to ensure that such research is carried out in accordance with the ethical principles set out in the Declaration of Helsinki and other guidelines.

### Products

For validation of the topography device, the products used were a disposable “cig-a-like” e-cigarette device (Vype Regular, Blue containing nicotine 3.0% v/v, glycerol and water), a rechargeable “cig-a-like” cartridge-based device (Vype Reload, Classic Flavour Bold, containing nicotine 4.5% v/v, glycerol and water) and a rechargeable, refillable tank-based device (Intellicig XL Pro with ECOpure Regular e-liquid, containing nicotine <3.2% v/v, glycerol and water). Flow rate measurements using the unmodified topography device used a disposable “cig-a-like” e-cigarette (Vype Regular, Red containing nicotine 4.5% v/v, glycerol and water).

For the puffing topography study, participants were assigned either a rechargeable “cig-a-like” device (Vype Reload, Classic Flavour Bold containing 4.5% v/v nicotine) or a larger, button-activated vaping product with two voltage settings (Vype ePen with a 3.0% v/v nicotine formulation). They were assigned the product that most closely resembled the type of e-cigarette they regularly used. Thus, users of smaller cartridge-based e-cigarettes were assigned Vype Reload and users of open, refillable vaping products were assigned Vype ePen. Each device was issued with a new cartridge and fully charged device battery. All devices and cartridges were supplied by Nicoventures Holdings Ltd., Blackburn, UK.

### Study Design

Participants made two visits to a central testing facility. At visit one, each participant was briefed on the study activities, assigned a unique identifier to be used throughout the study and asked to complete a questionnaire on demographic characteristics and product use history. At both visits, participants were required to use the assigned study product for a self-determined session length intended to reflect typical use, vaping on the product through a mouth piece connected to the modified SA7 topography head (see Supplementary Fig. S1). The participants were free to hold and move the topography device and e-cigarette within the constraints of the length of tubing (approximately 180 cm) connected to the data acquisition and transfer unit. Participants in the Vype ePen group used the product at the high and the low device voltage settings. One setting per visit was assigned by a staff member, in a randomised order, with the participant unaware of which was used.

### Topography Measures

The topography device used in this study was calibrated for pressure and flow rate on a daily basis, following the procedures described in the topography device validation section. For each participant’s session, the following data were recorded on a puff-by-puff basis: duration (s), volume (mL) and flow rate (mL/s). The number of puffs and the session length (min:s) were also recorded. Intervals between puffs were calculated after data collection.

### Data Preparation and Analysis

Individual puff volumes for air measured by the topography device during device validation were measured against a pre-set tolerance of ±1 mL, and those for e-cigarettes were against a larger pre-set tolerance of ±5 mL to allow for the effect of the aerosol on puff volume. Individual puff durations were compared against a pre-set tolerance of ±0.1 s.

The puff-by-puff topography data were averaged per replicate (study visit for Vype Reload and voltage for Vype ePen), and compared with paired *t* tests. Topography values for the two user groups were compared with a two-sample *t* test. Two tailed *P* values <0.05 were considered to be significant. The effect of length of time of historical e-cigarette use on mean puff duration was assessed by one-way ANOVA. Minitab V16 software (Minitab, Coventry, UK) was used to perform the data analysis.

## Results

### Topography Device Validation

Flow rate calibration across the range 2 mL/s to 120 mL/s resulted in all air puff volumes falling within the pre-set target tolerance of ±1 mL across the range of 20–80 mL (see Supplementary Fig. S2). Puff durations measured using the modified topography device showed excellent correlation to those measured by the unmodified SA7 topography device (see Supplementary Fig. S3). All puff volumes with e-cigarettes under the three puffing regimes were within 5 mL for all device types, with the largest deviation from the pre-set volume being 8.2% (4.5 mL; Table 1). Puff durations recorded by the modified topography device were within the pre-set target tolerance of ±0.1 s when compared with those recorded by the unmodified SA7 topography system. Initial puff volume determinations using a cleaned unmodified device were within ±0.7 mL across the range 25–100 mL, which increased to ±7.4 mL when repeated using the same topography head following use for 100 puffs on a disposable e-cigarette (see Supplementary Table S3).

### Characteristics of the Study Participants

We enrolled a total of 60 participants. The characteristics and product use history of the study participants are provided in Supplementary Table S4.

### Participants’ Puffing Topography Data

Participants’ puffing topography was assessed at two study visits for all participants except one in the Vype ePen group, for whom data were available at the high voltage setting only. We found no significant differences between study visits for Vype Reload (Table 2) or between voltage settings for Vype ePen (Table 3). Comparison between the two user groups showed statistically significant differences in the puffing topography attributes except for session length and mean puff duration (Table 4). Analysis of topography data by duration of e-cigarette use showed no significant difference in puff duration for either Vype ePen or the Vype Reload user group.

## Discussion

This study was designed to develop and validate a puffing topography device adapted for use with e-cigarettes and to measure users’ topography for two e-cigarette products. Changes to the topography device head were made to address the known limitations of puffing topography devices when used with e-cigarettes—condensation of aerosol, ability to measure only a limited number of puffs and reliable measurement at low flow rates. Our findings demonstrate the robustness of the modified topography head to measure puff volumes following repeated puffing (up to 150 puffs) on the e-cigarette devices. This development enables collection of reliable puffing topography data to be added to the scientific literature and will help in the development of standardised laboratory testing protocols for e-cigarettes that better reflect actual consumer behaviour.

Puffing topography devices have been extensively used to study smokers’ puffing behaviours, and several studies have reported little effect of the measurement process on puffing topography parameters and the smoking sensory experience. Blank *et al*.^9^ compared the effect of using direct observation measurements against those of portable and desktop puffing topography devices, and found relatively small differences between methods. Ross *et al*.^11^ reported no systematic differences in how cigarettes are smoked with topography devices relative to natural smoking. Lee *et al*^14^ investigated whether smokers changed their puffing behaviour over time when smoking through a puffing topography device and found no significant effect, although day to day variability led to 7% variance in topography measures. Those results demonstrate that topography measurements can yield exposure data that are representative of actual use.

The study by Blank *et al*.^9^ comparing video recording and puffing topography devices showed longer puff durations with video recordings than with topography devices. The increased puff durations with direct observation may have been caused by participants holding the cigarettes in their mouth before puffing, or keeping them there during the mouth-hold phase. Initial studies of the puffing behaviour of vapers involved analysis of video footage to measure puff durations. Hua *et al*.^15^ used a stopwatch to record puff durations, and found that those for e-cigarettes were significantly longer than those measured for smokers. Hua used the time the e-cigarette LED light was on to determine puff duration, which could limit the effect of keeping the device in the mouth. Farsalinos *et al*.^16^ used video-processing software to measure puff duration on a frame-by-frame basis. Results from this study agreed with those reported by Hua *et al*.

Some button-activated e-cigarette devices are available that offer the opportunity to approximate puffing duration by recording, within the e-cigarette, the length of time the user activates the heating element^12^,^13^. However, many users pre-heat their heating coils before puffing and, therefore, this approach can overestimate actual puff durations.

A small number of studies have used the commercially available CReSS puffing topography device (Borgwaldt) to obtain e-cigarette users’ puffing topography data, but researchers have reported reliability issues and data capture limitations. Norton *et al*.^17^, reported that device failure led to a loss of nine participants’ data. Behar *et al*.^18^ noted that although the CReSS devices were supplied with e-cigarette adaptors, no specific user instructions were available for use of the device with e-cigarettes, and 4–5 months of method development was necessary to be able to use the device reliably. In addition, the authors reported limitations of the device when collecting more than 43 puffs, leading to inaccurate puff number determinations in 26% of the user sessions. Other researchers have reported fewer issues with their own non-commercial devices^19^,^20^.

Spindle *et al*.^19^ reported two potential challenges to measuring e-cigarette users’ topography: first, condensation and build-up of aerosol within the topography device, and second, the ability of the device to measure low flow rates generated by e-cigarette users. The modifications we made to our topography head limited the susceptibility of the device to report inaccurate flow rates and puff volumes. Whilst initial measurements of puff volumes were successful using the unmodified device in a cleaned condition, the accuracy of the measurements decreased following repeated puffing on a disposable e-cigarette through the device (see Supplementary Table S3). Visible droplets of deposited aerosol were observed within the tubing of the topography device on prolonged usage with the e-cigarette and led to the development of the modified device to limit the effect of aerosol deposition. For the modified topography device, continual puffing on three different e-cigarette types to exhaustion of the device battery or a maximum of 150 puffs, under three puffing regimes, resulted in puff volumes within the range of pre-set tolerances. Whilst it is unlikely that an e-cigarette would be used from battery recharge to exhaustion or for 150 puffs during a single vaping session, the accuracy of the measured puff volumes provides evidence of the modified topography device’s suitability to be used for extended periods of time. The ability of the topography device to accurately measure low flow rates was tested during validation by inclusion of 33 calibration flow rates down to 2 mL/s. Ensuring this capability results in more of each individual puff being captured and, therefore, leads to increased accuracy of puff volume and duration measurements. Coupled with the device’s ability to record data at a sampling rate of every 40 ms, which results in a more precise measurement of the transient features of each individual puff, puffing topography datasets can be accurately duplicated in the laboratory to provide a measure of users’ exposure to aerosol emissions^22^.

The puffing topography parameters measured in this study fell within the range of values measured with puffing topography devices in the literature; mean puff volumes of 52.3 and 83.0 mL versus 51–133 mL and mean puff durations of 2.0 and 2.2 s versus 1.8-4.16 s^17^,^18^,^19^,^20^,^23^,^24^. Data from several sources have shown that for vaping products puff duration is the main determinant of the amount of aerosol per puff, whereas puffing volume and air flow speeds has little influence^5^. These effects are in sharp contrast to how cigarettes respond, and are thought to comprise the main reason why smokers adapt their puffing behaviour as they learn to vape.

Puff durations have been reported to be longer for e-cigarette users than cigarette smokers^12^,^15^,^16^, and for duration to increase over time with increasing e-cigarette experience^16^,^24^. However, the time frame involved in adaption appears to be relatively short. Lee *et al*.^24^, found significant increases in puff duration after 1 week of e-cigarette use by cigarette smokers, followed by a small decrease in the second week. We found no differences in mean puff duration between users who had been vaping for more than 6 months and those who had vaped for at least 1 month but not more than 6 months. It would thus appear that behaviour is largely stabilised within this first month of use. This interpretation would also be consistent with the change in mean ‘puff duration’, approximated by button activation time that has been recorded over 2 months from initiation of use of certain eGO type open tank products^12^. The mean puff duration increased from approximately 3.4 s to 4.1 s within the first 2 weeks of use of the product and then remained stable near that value for the next 6 weeks of study time. In that study users new to using that specific product may not have been new to vaping and, therefore, any adaptation effect would have been less pronounced. On the basis of the discussion above, we assume that the vapers in our study had largely adapted to vaping before participating.

Behar *et al*.^18^ observed significant differences in topography measures (except puff number) between two brands when used by the same participants on the same day, although, in absolute terms (mean puff volumes 56 mL versus 45 mL and durations of 2.75 s versus 2.54 s), the differences were smaller than those previously reported across multiple studies. The aerosol mass emissions from the button-activated product used here, Vype ePen, were significantly higher than those of the “cig-a-like” product, Vype Reload, under standardised machine testing conditions. The significant differences found in mean puff number, volume, interval and peak flow between devices supports the hypothesis that topography is not only determined by user characteristics, but also by product design. This theory is supported by the absence of differences in topography data between study visits for either of the two groups.

Additional data to support the ability of the topography device to measure puffing behaviours are those generated from the use of the unmodified SA7 topography device to measure puffing topography for smokers of a range of cigarette products^25^. Puff volumes in the range of 42.9 and 54.3 mL reported by Ashley *et al*.^25^ fall within the range of 30.8 to 67.5 mL reported by others for cigarette smokers^9^,^14^,^17^,^19^. Furthermore, larger puff volumes for e-cigarette users compared with smokers as measured by the same topography device have been reported by Norton *et al*.^17^, which agrees with the larger mean puff volume of 83 mL reported here for the users of Vype ePen compared to those reported for cigarette smokers^25^.

The wide variation observed across topography studies and devices highlights the challenges in establishing standardised laboratory testing protocols for e-cigarettes before the contributing factors are fully characterised. Contributing factors probably include differences between products (e.g. pressure drop, battery power and nicotine strength), accuracy of topography devices and participants’ use history and demographics. Whether study design parameters alter users’ natural behaviour should be considered when undertaking studies intended to reflect real-life use. For example, participants in the study by Spindle *et al*.^19^ were instructed to take 10 puffs with a fixed interval of 30 s, whereas Behar *et al*.^18^ limited session lengths to 10 min. Both of these designs do not reflect real-world vaping use, and the impact of any of these restrictions on study findings is unclear.

A number of limitations apply to this study. First, the participants were using unfamiliar products, and features such as the nicotine strength and formulation of the e-liquid which varied across the study products might have affected puffing behaviour, but reflects that more powerful e-cigarette devices typically use lower concentrations of nicotine. Future research should focus on participants using their normal product, which could allow a systematic study design to be used thus allowing for the effects of factors such as device type and nicotine strength on puffing topography to be studied. In addition future studies should consider the impact of participants’ characteristics such as dual usage of cigarettes and e-cigarettes on topography data. Second, having to attend a central location for testing and being in the presence of research staff could have shortened session lengths and led to puffs with reduced intervals. Portable topography devices that capture data over a number of usage sessions within a 24 h period or number of days would provide more naturalistic user data^17^,^20^ but at the possible expense of reduced data integrity.

## Conclusion

The puffing topography device we have developed accurately measured all features of e-cigarette puff topography and overcame some of the challenges and limitations reported with other methods, namely, indirect determination of puff durations, deposition of aerosol within the topography device affecting volume measurements and reliable measurements at low flow rates. No differences were found between replicates within either of the groups tested. Differences were found between users of the two study products for the majority of the topography measures. To develop standardised testing protocols for e-cigarettes, being able to accurately duplicate naturalistic puffing topography will be of key importance. Furthermore, systematically designed studies are required to understand the factors that influence users’ puffing topography data. This will enable better understanding of consumer behaviour and provide data on users’ exposure that are closer to real-life use in the everyday environment.

## Additional Information

**How to cite this article**: Cunningham, A. *et al*. Development, validation and application of a device to measure e-cigarette users’ puffing topography. *Sci. Rep.* **6**, 35071; doi: 10.1038/srep35071 (2016).

## Supplementary Material

Supplementary Information

## Figures and Tables

**Table 1:** Comparison of measured to set puff volumes recorded in the laboratory studies using three e-cigarette types.

Device	Pre-Set Puff Volume (mL)^a^		
	55	80	120
Disposable	n = 267	n = 266	n = 234
	55.1 (0.2)	80.5 (0.3)	120.0 (1.1)
Rechargeable cartomiser-based	n = 434	n = 399	n = 423
	54.8 (0.2)	80.0 (0.3)	119.1 (0.6)
Rechargeable modular/Tank system	n = 450	n = 451	n = 452
	55.0 (0.8)	80.4 (1.2)	120.0 (1.2)

^a^Presented as mean values (standard deviation).

**Table 2:** Mean (standard deviation) puffing topography data for Vype Reload user group.

Parameter	Replicate 1 (n = 32)	Replicate 2 (n = 32)	All replicates	*P* value (replicate 1 vs replicate 2)
Session length (min:s)	7:10 (4:12)	6:38 (3:13)	6:54 (3:43)	0.372
Puff number (n)	22.0 (16.3)	20.3 (13.6)	21.1 (14.9)	0.588
Mean puff volume (mL)	50.4 (21.0)	54.0 (22.4)	52.2 (21.6)	0.079
Mean puff duration (s)	2.0 (0.6)	2.1 (0.7)	2.0 (0.7)	0.393
Mean inter-puff interval (s)	22.8 (10.8)	23.6 (10.7)	23.2 (10.6)	0.717
Mean peak flow-rate (mL/s)	38.0 (10.2)	39.9 (10.4)	39.0 (10.3)	0.144

**Table 3:** Mean (standard deviation) puffing topography data for Vype ePen user group.

Parameter	High voltage (n = 28)	Low voltage (n = 27)	All measurements	*P* value (high voltage vs low voltage)
Session length (min:s)	7:05 (5:33)	8:19 (7.00)	7:41 (6:17)	0.409
Puff number (n)	14.3 (6.0)	18.1 (9.3)	16.1 (8.0)	0.067
Mean puff volume (mL)	83.8 (42.5)	82.1 (46.9)	83.0 (44.3)	0.908
Mean puff duration (s)	2.2 (0.9)	2.1 (0.9)	2.2 (0.9)	0.364
Mean inter-puff interval (s)	30.1 (19.6)	28.5 (19.0)	29.3 (19.2)	0.596
Mean peak flow-rate (mL/s)	60.5 (21.1)	60.7 (18.8)	60.6 (19.8)	0.757

**Table 4:** Mean (standard deviation) puffing topography data by user group.

Parameter	Vype Reload (n = 64)	Vype ePen (n = 55)	*P* value (Reload vs ePen)
Session length (min:s)	6:54 (3:43)	7:41 (6:17)	0.417
Puff number (n)	21.1 (14.9)	16.1 (8.0)	0.022
Mean puff volume (mL)	52.2 (21.6)	83.0 (44.3)	0.000
Mean puff duration (s)	2.0 (0.7)	2.2 (0.9)	0.382
Mean inter-puff interval (s)	23.2 (10.6)	29.3 (19.2)	0.039
Mean peak flow-rate (mL/s)	39.0 (10.3)	60.6 (19.8)	0.000
