# Supplementary material for: Development, validation and application of a device to measure e-cigarette users’ puffing topography
Source: Sci Rep. 2016 Oct 10;6:35071. doi: 10.1038/srep35071 (PMC5056340; doi:10.1038/srep35071)
Supplement: Supplementary Information [file srep35071-s1.doc]

**Development, validation and application of a device to measure e-cigarette users’ puffing topography**

Anthony Cunningham, Sandra Slayford, Carl Vas, Jodie Gee, Sandra Costigan, Krishna Prasad

**Supplementary Table S1. Regimes used to test accuracy of puff volumes** and durations measured using the modified topography head

| **Regime** | **Puff volume (mL)** | **Puff durations (s)** |
| --- | --- | --- |
| 1 | 20 | 1.5 |
| 2 | 40 | 2.0 |
| 3 | 60 | 2.5 |
| 4 | 80 | 3.0 |

**Supplementary Table S2. Regimes used to test accuracy of puff volumes from three e-cigarettes types**

| **Regime** | **Puff volume (mL)** | **Puff duration (s)** | **Puff interval (s)** | **Average flow rate (mL/s)** |
| --- | --- | --- | --- | --- |
| 1 | 55 | 3.0 | 30 | 18.3 |
| 2 | 80 | 3.0 | 30 | 26.7 |
| 3 | 120 | 3.0 | 30 | 40.0 |

**Supplementary Table S3. Puff volume determinations using a clean unmodified topography device and repeated following 100 puffs on disposable e-cigarette using a regime of 80mL volume, 3s duration and 30 s interval**

| **Pre-set puff volume (mL)** | **Initial measured puff volume (mL)** | **Measured puff volume post 100 puffs (mL)** |
| --- | --- | --- |
| 25 | 25.0 | 26.2 |
| 50 | 49.9 | 52.8 |
| 75 | 75.1 | 79.9 |
| 100 | 100.4 | 107 |
| 25 | 24.8 | 26.1 |
| 50 | 50.2 | 52.8 |
| 75 | 75.1 | 79.3 |
| 100 | 99.9 | 106.3 |
| 25 | 25 | 26.6 |
| 50 | 50.2 | 53.3 |
| 75 | 75.4 | 80.2 |
| 100 | 100.7 | 107.4 |
| 25 | 25.0 | 26.5 |
| 50 | 50.2 | 53.1 |
| 75 | 75.2 | 80.1 |
| 100 | 100.4 | 107.3 |

**Supplementary Table S4. Characteristics of the study participants and product use history**

| **Variable** |  | **All Volunteers (n=60)** | **Vype Reload User Group (n=32)** | **Vype ePen User Group (n=28)** |
| --- | --- | --- | --- | --- |
| Gender | Male | 32 | 16 | 16 |
|  | Female | 28 | 16 | 12 |
| Age^a^ | 18-24 | 5 | 4 | 1 |
|  | 25-34 | 23 | 10 | 13 |
|  | 35-44 | 18 | 10 | 8 |
|  | 45+ | 13 | 8 | 5 |
|  | Mean Age (SD) | 36.4 (10.6) | 37.5 (12.1) | 35.1 (8.6) |
| Duration of e-cigarette use | 1-2 months | 12 | 5 | 7 |
|  | 3-6 months | 22 | 10 | 12 |
|  | 6+ months | 26 | 17 | 9 |
| Average use per day of e-cigarette | Once | 13 | 8 | 5 |
|  | 2-3 times | 20 | 14 | 6 |
|  | 4 or more | 27 | 10 | 17 |

^a^ One age value not provided for participant in ePen user group

Spigot adapter to attach e-cigarettes. Spigot removes jetting through orifice, which results in inaccurate puff volumes


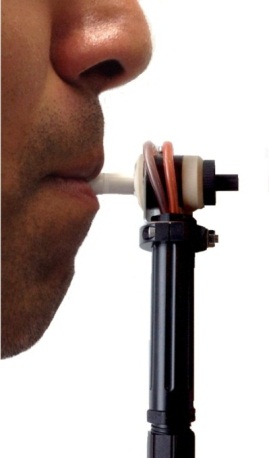


Pressure ports on top to reduce excipients from blocking tubes


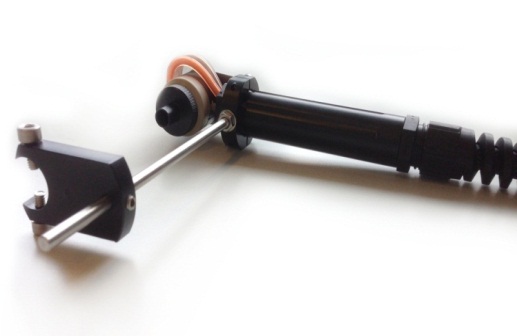


Bracket to support larger

e-cigarette modular devices

Removable cap allows access to orifice plate for cleaning


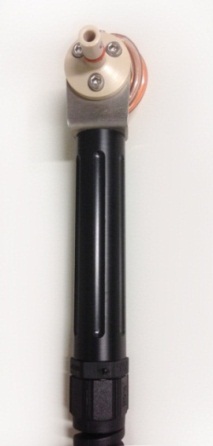


**Supplementary Figure S1. Modifications made to the topography device head**


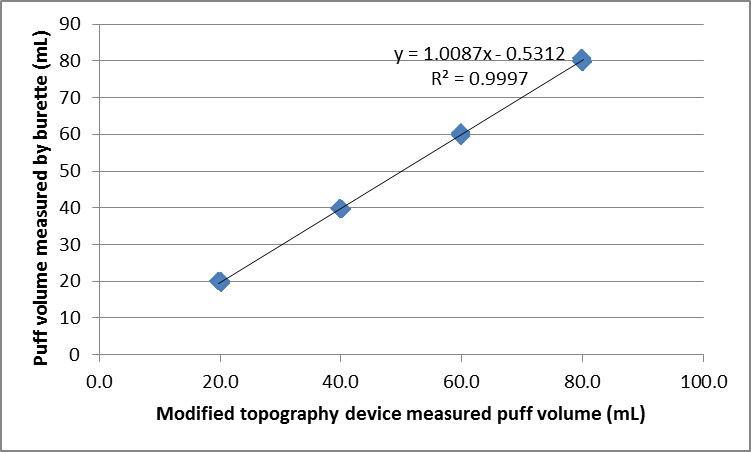


**Supplementary Figure S2. Accuracy of puff volume measurements of modified topography device compared with burette**


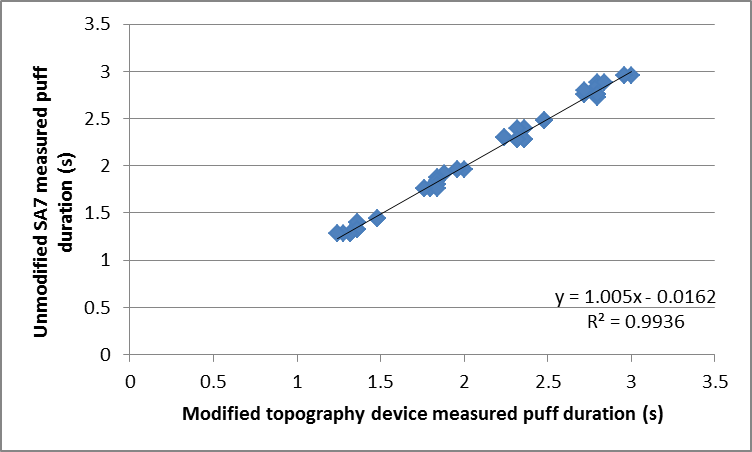


**Supplementary Figure S3. Accuracy of puff duration measurements of modified topography device compared with unmodified SA7 topography device**
